# Association of Organizational Pathways With the Delay of Emergency Surgery

**DOI:** 10.1001/jamanetworkopen.2023.8145

**Published:** 2023-04-13

**Authors:** Delphine Lepercq, Tobias Gauss, Anne Godier, Julie Bellet, Guillaume Bouhours, Pierre Bouzat, Emeline Cailliau, Fabrice Cook, Jean-Stéphane David, Fatou Drame, Marvin Gauthier, Antoine Lamblin, Julien Pottecher, Benoit Tavernier, Delphine Garrigue-Huet

**Affiliations:** 1 Pôle d’anesthésie-réanimation, CHU de Lille, Lille, France; 2 Division of Anesthesia–Critical Care, Grenoble Alpes University Hospital, Grenoble, France; 3 Department of Anaesthesiology and Critical Care, AP-HP, Hôpital Européen Georges Pompidou, Paris, France; 4 Université de Paris, Inserm, Innovations Thérapeutiques en Hémostase, Paris, France; 5 Département Anesthésie Réanimation, Centre Hospitalier Universitaire d’Angers, Angers, France; 6 University of Grenoble Alpes, Inserm, U1216, Grenoble Institut Neurosciences, Grenoble, France; 7 Biostatistics Department, CHU Lille, Lille, France; 8 Department of Anesthesia and Surgical Intensive Care, Henri Mondor University Hospital of Paris, Paris XII School of Medicine, Creteil, France; 9 Service d’Anesthésie Réanimation, Groupe Hospitalier Sud, Hospices Civils de Lyon, Lyon, France; 10 AP-HP, Beaujon University Hospital, DMU PARBOL, Department of Anaesthesiology and Critical Care, Clichy, France; 11 Anesthesiology and Critical Care Medicine Department, Edouard Herriot Hospital, Hospices Civils de Lyon, Lyon, France; 12 Espace Ethique Méditerranéen, Efaculté de Médecine de Marseille, Timone University Hospital, Marseille, France; 13 Hôpitaux Universitaires de Strasbourg, Hôpital de Hautepierre, Department of Anaesthesiology, Critical Care and Perioperative Medicine, Université de Strasbourg, Faculté de Médecine, Fédération de Médecine Translationnelle de Strasbourg, ER 3072, Strasbourg, France; 14 Université Lille, CHU Lille, ULR 2694–METRICS, Lille, France

## Abstract

**Importance:**

Delayed admission of patients with surgical emergencies to the operating room occurs frequently and is associated with poor outcomes. In France, where 3 distinct organizational pathways in hospitals exist (a dedicated emergency operating room and team [DET], a dedicated operating room in a central operating theater [DOR], and no dedicated structure or team [NOR]), neither the incidence nor the influence of delayed urgent surgery is known, and no guidelines are available to date.

**Objective:**

To examine the overall frequency of delayed admission of patients with surgical emergencies to the operating room across the 3 organizational pathways in hospitals in France.

**Design, Setting, and Participants:**

This prospective multicenter cohort study was conducted in 10 French tertiary hospitals. All consecutive adult patients admitted for emergency surgery from October 5 to 16, 2020, were included and prospectively monitored. Patients requiring pediatric surgery, obstetrics, interventional radiology, or endoscopic procedures were excluded.

**Exposures:**

Emergency surgery.

**Main Outcomes and Measures:**

The main outcome was the global incidence of delayed emergency surgery across 3 predefined organizational pathways: DET, DOR, and NOR. The ratio between the actual time to surgery (observed duration between surgical indication and incision) and the ideal time to surgery (predefined optimal duration between surgical indication and incision according to the Non-Elective Surgery Triage classification) was calculated for each patient. Surgery was considered delayed when this ratio was greater than 1.

**Results:**

A total of 1149 patients were included (mean [SD] age, 55 [21] years; 685 [59.9%] males): 649 in the DET group, 320 in the DOR group, and 171 in the NOR group (missing data: n = 5). The global frequency of surgical delay was 32.5% (95% CI, 29.8%-35.3%) and varied across the 3 organizational pathways: DET, 28.4% (95% CI, 24.8%-31.9%); DOR, 32.2% (95% CI, 27.0%-37.4%); and NOR, 49.1% (95% CI, 41.6%-56.7%) (*P* < .001). The adjusted odds ratio for delay was 1.80 (95% CI, 1.17-2.78) when comparing NOR with DET.

**Conclusions and Relevance:**

In this cohort study, the frequency of delayed emergency surgery in France was 32.5%. Reduced delays were found in organizational pathways that included dedicated theaters and teams. These preliminary results may pave the way for comprehensive large-scale studies, from which results may potentially inform new guidelines for quicker and safer access to emergency surgery.

## Key Points

**Question:**

What is the global frequency of delayed management of surgical emergencies in France?

**Findings:**

In this cohort study of 1149 patients, the frequency of delayed emergency surgery was 32.5% and varied substantially across 3 distinct organizational pathways of care: dedicated emergency surgery department and team, dedicated emergency operating room, and no dedicated emergency operating room or team.

**Meaning:**

This study demonstrated a substantial global frequency of delayed emergency surgery in France, with large differences depending on the organizational pathway.

## Teaser

This cohort study examines rates of delayed emergency surgery in French hospitals that have a dedicated emergency surgery department and team, a dedicated emergency operating room, or no dedicated emergency operating room or team.

## Introduction

Emergency surgery represents a significant and increasing proportion of operating activity in institutions across the world and is even considered a specialty in some countries.^1,2^ In the US, more than 3 million patients are admitted each year for urgent abdominal surgery, and the associated costs are increasing.^1,3^

As opposed to elective care, emergencies are characterized by reduced preoperative time for comprehensive patient workup optimization and team coordination. Emergency surgery is associated with a higher risk of mortality and postoperative complications.^4,5,6^ Additionally, organizational issues, such as staff unavailability, frequently delay emergency surgery. Delayed emergency surgery is associated with increased risk of adverse events and complications.^7,8,9,10^ McIsaac et al^11^ studied delayed access to surgery in a retrospective Canadian cohort in 2017. Among 15 000 emergency patients, 3000 underwent surgery with delay (20%); this delay was associated with higher in-hospital mortality and length and cost of stay. The proportion of delayed surgical emergencies is a benchmark proxy to investigate the performance of the health care system. To address the challenge of timely access of emergent surgical cases to the operating room, dedicated multidisciplinary standing operating teams and specific classifications for emergency surgery facilitate the prioritization between urgent and nonurgent cases.^12,13^ Dedicated teams and risk stratification networks optimize the use of resources,^14,15,16,17,18^ reducing the delay to emergency surgery and complications.

In France, the incidence of delayed emergency surgery is unknown, and no guidelines are available to date. Currently, 3 types of care pathways for emergency surgery predominate in France: (1) an exclusive emergency theater with a dedicated emergency operating theater and team (DET); (2) 1 (or more) dedicated emergency operating room in a shared, all-purpose, multidisciplinary surgical theater (DOR); and (3) no dedicated emergency operating room (NOR), in which emergency cases are integrated into the elective surgery workflow (absence of a dedicated pathway) (eFigure 1 in Supplement 1).

In consequence, it appeared necessary to study the frequency of delayed emergency surgery depending on the 3 aforementioned organizational pathways in France. Based on the international experience, we hypothesized that the DET pathway would be associated with reduced incidence of delay and shorter time to surgery.^14,19,20,21,22,23,24^

## Methods

### Study Design and Patients

This prospective, multicenter cohort study took place in 10 French tertiary academic centers (eAppendix 1 in Supplement 1) and followed the Strengthening the Reporting of Observational Studies in Epidemiology (STROBE) reporting guideline. The Comité de Protection des Personnes Nord Ouest IV approved the study and waived informed consent, considering written information of all patients as sufficient and in agreement with French legislation.^25^ All data sets were registered with Lille University Hospital’s data protection officer.

During the study period, all consecutive adult patients admitted for emergency surgery and with social security coverage were included from October 5 to 16, 2020. Urgent surgery was defined as a condition requiring surgery within 72 hours. Patients requiring pediatric surgery, obstetrics, endoscopy, or interventional radiology were excluded, as were patients under guardianship. Patients enrolled in prospective studies were not recruited.

In all participating centers, data were prospectively retrieved from electronic patient records, the emergency department, anesthetic records, electronic imaging systems, and operative reports. Patients were monitored until their 30th postoperative day.

### Outcomes and Exposures

The main outcome was the global frequency of delayed admission to the operating room for patients requiring urgent surgery. To determine this delay, 2 surgical times were labeled: the observed time, or the actual time to surgery (aTTS), and the ideal time to surgery (iTTS). The aTTS was the time observed between the decision in favor of an unequivocal, definitive need for surgery (surgical indication), for example, after failure of a medical treatment or observation. This point in time was indicated by the surgeon responsible for the surgery. In consequence, the aTTS was the time between surgical indication and incision in the operating theater. The iTTS was the predefined optimal time between surgical indication and incision according to the Non-Elective Surgery Triage (NEST) classification. The NEST classification facilitates prioritization of surgical cases (eTable 1 in Supplement 1)^17^ and comprises 6 decreasing levels of urgency (from 1, the most urgent, to 6, the least urgent). Each NEST category is defined by an iTTS. A NEST class of 1 corresponds to life-saving surgery and a NEST class of 6 to interventions to be performed within 72 hours. In consequence, any case was considered as delayed and accounted for the primary outcome whenever the ratio of aTTS to iTTS was greater than 1 (eFigure 2 in Supplement 1).

All of the most frequently performed emergency surgeries at Grenoble Alpes University Hospital and Lille University Hospital were classified by a college of experienced surgeons from both institutions according to NEST criteria to determine the iTTS and identify interventions not covered by NEST. For surgeries not covered by NEST, the college defined an iTTS. The complete listing of surgical emergencies with their respective iTTS was provided to each participating center to serve as a template to calculate the delay (eAppendix 2 in Supplement 1). If a case with no predefined iTTS was included, the attending surgeon determined the iTTS to the best of her or his abilities. For each patient, the ratio was calculated using the iTTS or, if not available, the surgeon-determined iTTS.

Additionally, 5 clinical severity criteria (infectious, hemorrhagic, ischemic, neurological, and multiple trauma) (eAppendix 3 in Supplement 1) were used to assess the surgical urgency of the patient’s clinical condition (eFigure 3 in Supplement 1). The severity criteria were used to upgrade patients.^26^ These criteria were applied after coordination between the attending surgeon and anesthetist at their discretion before admission to the operating room.

The secondary objectives consisted of the frequency and importance of delay (according to the aTTS to iTTS ratio) for each organizational pathway independent of age, sex, clinical severity criteria, American Society of Anesthesiologists (ASA) physical status classification (range, 1-6, with higher scores indicating more severe systemic disease and functional impairment),^27^ and NEST classification; intrahospital mortality and postoperative complications according to the Clavien-Dindo classification (class II to V) (eAppendix 4 in Supplement 1); and the association of delay with morbidity and mortality, surgical workflow, and staff organization. The proxies for the association of delay with surgical workflow corresponded to the number of cancellations, rescheduling of elective or nonelective surgery, and secondary transfers of patients to hospitals with available operating capacities. The proxy to assess the association of any delay with staff organization corresponded to supplementary activation of staff and any increase in overtime. Causes of delay were classified into patient-specific and organizational causes. The COVID-19 pandemic resulted in a specific cause of delay: “waiting for COVID test results.” Follow-up ended on day 30.

### Statistical Analysis

Categorical variables were expressed as frequencies and percentages. Quantitative variables were expressed as means and SDs or medians and IQRs. Normality of distribution was verified graphically and with the Shapiro-Wilk test. The delay rate was calculated with its 95% CI and compared across the 3 organizational pathways using a multivariable logistic regression model adjusted for predefined confounding factors: age, sex, clinical severity criteria, ASA classification, and NEST classification (theoretical NEST or the surgeon’s NEST). Based on McIsaac et al,^11^ the expected frequency of delay was approximately 18%. The investigators anticipated to estimate the theoretical frequency with a precision of 2%, which relates to half of the 95% CI. To obtain this precision, the required sample size was 1500 patients. Associations of delay with intrahospital mortality and postoperative complications were estimated using a multivariable logistic regression model adjusted for the predefined confounding factors and the organizational pathway. Odds ratios (ORs) were estimated with their 95% CIs. Length of hospital stay was estimated by the Kalbfleisch and Prentice method^28^ to consider death as a competing event and compared between delayed and not delayed operations using a Fine and Gray competing risk regression model adjusted for the predefined confounding factors and the organizational pathway. The hazard ratio was estimated with its 95% CI. Associations of delay and pathway with surgical workflow and staff organization were estimated using a multivariable logistic regression model including delay, pathway, and the predefined confounding factors as covariates. Statistical testing was conducted at a 2-tailed significance level of *P* < .05. Data were analyzed using SAS software, version 9.4 (SAS Institute).

## Results

### Population

**Figure 1.**
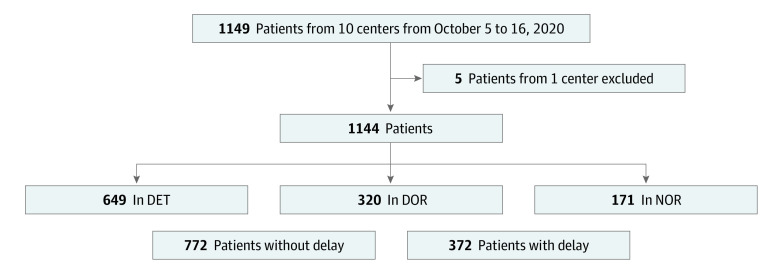
Flowchart of the Study DET indicates dedicated emergency theater and team; DOR, dedicated emergency operating room; and NOR, no dedicated emergency operating room or team.

In total, the study recruited 1149 patients; 5 patients (0.4%) were excluded because their center dropped out of the study. Among the remaining 1144 patients, the mean (SD) age was 55 (21) years; 459 (40.1%) were female, 685 (59.9%) were male, and 418 (36.5%) had ASA class III or IV. A total of 649 (56.7%) were in the DET group, 320 (28.0%) in the DOR group, and 171 (15.3%) in the NOR group. Figure 1 shows the flowchart of the study.

The epidemiological, clinical, and surgical profiles together with the NEST classification of the included patients are reported in eTable 2 in Supplement 1. A predefined iTTS was appropriate for only 799 of the 1144 surgeries (69.8%). Therefore, time to surgery was calculated using an iTTS determined by the attending surgeon in the remaining 345 cases (30.2%) (eTable 2 in Supplement 1).

### Primary and Secondary Outcomes

**Figure 2.**
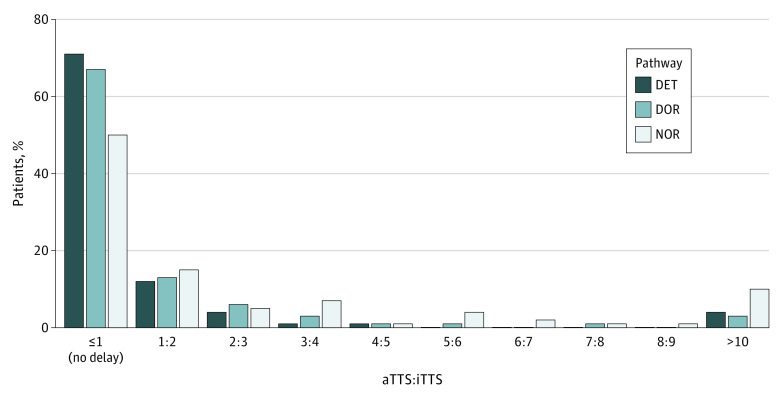
Frequency and Importance of Delay in Dedicated Emergency Theater and Team (DET), Dedicated Emergency Operating Room (DOR), and No Dedicated Emergency Operating Room (NOR) Pathways aTTS:iTTS indicates ratio of actual time to surgery to ideal time to surgery.

**Figure 3.**
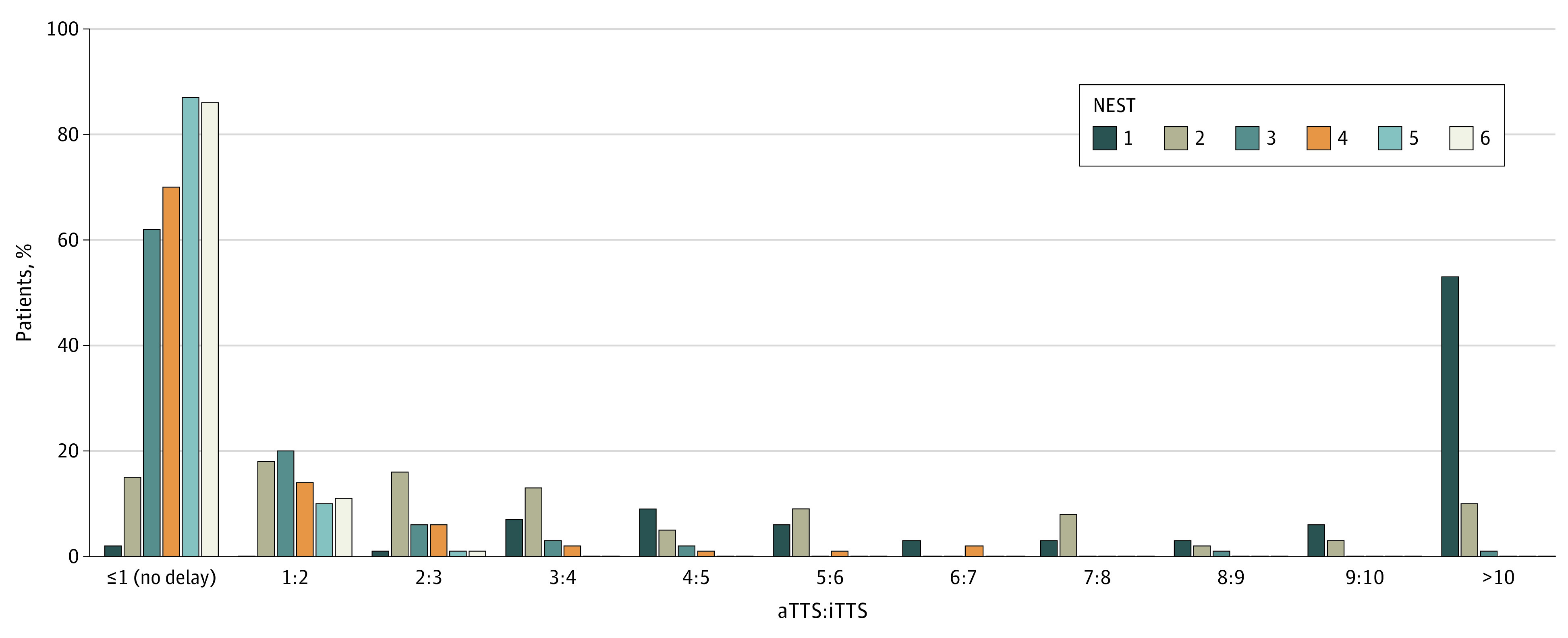
Actual Time to Surgery (aTTS) to Ideal Time to Surgery (iTTS) Ratio per Non-Elective Surgery Triage (NEST) Category The NEST classification comprises 6 decreasing levels of urgency (from 1, the most urgent, to 6, the least urgent).

The global frequency of delayed admission to the operating room for patients requiring emergency surgery (primary outcome) was 372 of 1144 cases (32.5%; 95% CI, 29.8%-35.3%). Figure 2 shows the frequency and importance of delay as assessed by the aTTS to iTTS ratio for each organizational pathway. The frequency of delay differed between pathways (28.4% [95% CI, 24.8%-31.9%] in DET, 32.2% [95% CI, 27.0%-37.4%] in DOR, and 49.1% [95% CI, 41.6%-56.7%] in NOR; *P* < .001) even after adjustment for confounding variables. The ORs of delay were 1.80 (95% CI, 1.17-2.78) for NOR vs DET and 0.91 (95% CI, 0.62-1.32) for DOR vs DET. Patients stratified to a NEST classification of 1 or 2 experienced more delays than patients stratified to a NEST class of 5 or 6 (OR, 54.78; 95% CI, 28.13-106.69; *P* < .001) (Figure 3); this result was similar for patients stratified to NEST class 3 or 4 vs NEST class 5 or 6 (OR, 2.54; 95% CI, 1.71-3.79; *P* < .001) (Figure 3).

The unadjusted complication rate was 29.7% (95% CI, 26.2%-33.3%) in the DET group, 27.6% (95% CI, 22.6%-32.5%) in the DOR group, and 40.9% (95% CI, 33.5%-48.4%) in the NOR group. The unadjusted mortality rate was 14.1% (95% CI, 11.4%-16.9%) for DET, 16.8% (95% CI, 12.6%-21.0%) for DOR, and 27.4% (95% CI, 20.6%-34.2%) for NOR.

After adjustment for confounding factors, the rates of intrahospital mortality and postoperative complications (Clavien-Dindo class II-V) did not differ significantly between the patients who underwent surgery with a delay and those who underwent surgery without delay (with delay: OR, 1.09 [95% CI, 0.65-1.83]; adjusted *P* = .75; without delay: OR, 1.09 [95% CI, 0.76-1.56]; adjusted *P* = .62). The length of hospital stay did not differ (OR for the risk of hospital discharge: 0.94; 95% CI, 0.79-1.12; adjusted *P* = .49).

Table 1 summarizes the analysis of the association of delayed emergency surgery and the pathway with overall surgical workflow (canceled or rescheduled elective surgery, transfer to another theater or hospital). Delayed interventions were associated with a higher risk of rescheduled or canceled elective surgery (adjusted OR, 2.27; 95% CI, 1.51-3.40). The DOR pathway was associated with a lower risk of influence on surgical flow compared with DET (adjusted OR, 0.58; 95% CI, 0.38-0.87). No difference was shown between DET and NOR concerning surgical workflow. The investigation did not find a measurable association of delay and surgical pathway with staff organization.

**Table 1.** Association of Interventions With Impaired Surgical Workflow^a^

	Interventions, No./total No. (%) (N = 192)	OR (95% CI)^b^	*P* value
Delayed			
No	92/767 (12.0)	1 [Reference]	<.001
Yes	100/372 (26.9)	2.27 (1.51-3.40)	
Pathway			
DET	119/649 (18.3)	1 [Reference]	.03
DOR	42/320 (13.1)	0.58 (0.38-0.87)	
NOR	31/171 (18.1)	0.80 (0.50-1.29)	

Abbreviations: DET, dedicated emergency theater and team; DOR, dedicated emergency operating room; NOR, absence of a dedicated emergency operating room; OR, odds ratio.

^a^ Impaired workflow included canceled or rescheduled surgery or transfer to other theater or hospital.

^b^ Odds ratios were calculated for operations that modified the surgical flow vs those that did not and were adjusted on predefined confounding variables (age, sex, clinical severity criteria, American Society of Anesthesiologists physical status classification, and Non-Elective Surgery Triage score).

Table 2 shows the results of analysis for the association of delayed emergency surgery and the organizational pathway with staff organization (additional staff for backup, overtime). No difference was shown for any of the 3 pathways.

**Table 2.** Association of Interventions With Impaired Staff Organization, Including Backup and Overtime

	Interventions, No./total No. (%)	OR (95% CI)^a^	*P* value
Delayed (n = 208)			
No	111/767 (14.5)	1 [Reference]	.38
Yes	97/372 (26.1)	1.20 (0.80-1.80)	
Pathway (n = 207)			
DET	119/649 (18.3)	1 [Reference]	.054
DOR	64/320 (20.0)	0.98 (0.68-1.41)	
NOR	24/171 (14.0)	0.54 (0.32-0.90)	

Abbreviations: DET, dedicated emergency theater and team; DOR, dedicated emergency operating room; NOR, absence of a dedicated emergency operating room or team; OR, odds ratio.

^a^ Odds ratios were calculated for operations that modified the staff organization vs those that did not and were adjusted on predefined confounding factors: age, sex, clinical severity criteria, American Society of Anesthesiologists physical status classification, and Non-Elective Surgery Triage score.

Organizational issues accounted for 188 of 372 delayed admissions (50.5%) and concerned mainly the availability of operating rooms and clinical staff. Organizational issues were not significantly associated with any of the 3 surgical pathways. Issues relating to patient management (additional examinations, SARS-CoV-2 test results, and medical optimization of the patient) were more frequent (235 of 372 [63.2%]). In 8 cases (2.2%), delay resulted from a combination of patient management and organizational issues.

## Discussion

This prospective cohort study showed a global frequency of delayed emergency surgery in France of 32.5% across 3 distinct organizational pathways. The difference was significant between the DET (28.4%) and NOR (49.1%) pathways. Complications and mortality were not different between patients who underwent surgery with or without delay, but the study was not powered to show such differences. The strength of the present study consists of the multicenter assessment of delay across 3 distinct organizational pathways across surgical specialties.

The observed frequency of surgical delay was higher than the rate described by McIsaac et al.^11^ Those authors did not explore organizational pathways. Restricting the comparison between the data from McIsaac et al^11^ and the current study’s DOR pathway demonstrated a greater rate of delay in the present study (32.2%) vs 18.6% in the other study. Of note, the study by McIsaac et al^11^ excluded cases with a delay exceeding the iTTS by 3 times and, in consequence, may have underestimated overall delay. In comparison, Schneider et al^7^ observed a delay in 36.9% of cases exploring a DOR pathway. Schneider et al^7^ included exclusively patients with emergency laparotomy, which may not allow a direct comparison with the present study.

In the present study, the most urgent cases were often surgically treated with delay. This compares unfavorably with a study from Koivukangas et al^29^ that showed that a higher degree of urgency was associated with increased chance for the patient to undergo surgery in a timely manner. However, Koivukangas et al^29^ defined a 3-hour time frame for the most urgent cases. In the present study, patients in the NEST 1 category were supposed to undergo surgery within 30 minutes. This time frame is challenging even for dedicated and trained teams. Patients in the NEST 1 or 2 category were often clinically unstable; in consequence, their condition sometimes required stabilization to perform the surgery safely, leading to a long delay. An important proportion of delays concerned aTTS to iTTS ratios of 1 to 2 or 2 to 3 (Figure 3). These delays may appear minor or may in part be explained by clinical reasoning and due process. The aTTS-iTTS method attempted to reduce any delay induced by observation and waiting for a medical treatment to work. Figure 3 also shows that the delays concerned mainly cases in NEST categories 1 and 2, for which small delays can induce considerable clinical impact.

To reduce the risk for delay, some teams have suggested the use of dedicated pathways for specific indications such as emergency laparotomy.^30^ In 1 study, time to surgery was reduced if the patient was examined at admission by an emergency surgeon.^31^ In the US, emergency surgery is a surgical specialty with specific training; this emphasizes the importance of a dedicated team.^2,32,33,34,35,36^ In the study by McIsaac et al,^11^ more urgent operations (41.4%), lack of staff (31.7%), and the need for patient examination or care (13.6%) were the main causes of delay. Cosgrove et al^19^ documented the unavailability of a surgeon as the main cause of delay (15.6%).

In terms of organizational factors, the NOR pathway was associated with an increased delay compared with the DET pathway. Other groups observed the same phenomenon after implementation of a DET in their institutions.^14,37,38^ Wanis et al^14^ documented a reduction in time to surgery from 3.7 to 3.2 hours (*P* = .02) and Sarmiento Altamirano et al^37^ from 10.6 to 3.2 hours in general surgery and 6.3 to 1.6 hours in traumatology (*P* < .05). In another study, a DET pathway was associated with increased operating room occupation from 57% to 69% (*P* < .001) and reduced nighttime occupation by 26% (*P* = .007), improving working conditions.^39^

The available evidence suggests that in addition to dedicated pathways, individualized risk assessment and triage and active, dedicated fast-track pathways are associated with improved management of high-risk patients based on clinical score.^26,40^ The advantage of a specific score would facilitate communication between all involved clinicians. Existing mortality prediction tools do not seem well suited since they do not account for the aforementioned causes of delay.^41^ Such a score should be a future avenue of research.

The present study did not demonstrate any measurable association of delay with either intrahospital mortality or postoperative complications, probably due to a lack of power and inappropriate design. Morbidity and mortality rates were higher than those observed by McIsaac et al^11^ but similar to those in other studies.^6,42^ Previous studies concluded that a DET pathway was associated with reduced complications, mortality, and length and cost of stay.^20,21,39,43^

### Limitations

In terms of limitations, the number of patients included (n = 1144) did not reach the initial objective (n = 1500). This was likely due to the difficulty to predict the number of patients undergoing surgery in each center and to 1 center dropping out. This limitation did not prevent the main objective from being achieved, and a higher incidence of delay than expected was found when exploring the 3 distinct pathways. The time to surgery assessed by the surgeon was not always in agreement with the iTTS proposed by the experts for the study. Often, surgeons considered the case to be more urgent than the categorization suggested. In the literature, the definitions of iTTS and delay to surgery vary among authors, which limits the reliability of comparisons between studies. As with any classification, the NEST categorization of a single patient into one or another category results from an arbitrary clinical gestalt. Categorization into a high-priority class but delayed surgery may not necessarily translate into adverse clinical consequences. This circumstance applies to any classification and is not specific to the NEST system. The investigators considered international comparability as a priority.^44,45,46^

## Conclusions

In this cohort study, the global frequency of delayed admission to the operating room for patients requiring emergency surgery was 32.5% for 3 distinct surgical pathways and across different surgical specialties. There seemed to be an advantage to reduced delay with a dedicated pathway either with a DET or DOR compared with NOR. Reduced delay to emergency surgery may be associated with improved patient outcomes and may facilitate appropriate resource use and allocation. The present results require confirmation in a large, multicenter study to inform national guidelines.

## Notes

Supplement 1. **eAppendix 1.** List of 9 French Centers **eTable 1.** The Non-Elective Surgery Triage (NEST) Classification **eAppendix 2.** iTTS of Surgery **eAppendix 3.** Severity Criteria **eAppendix 4.** Clavien-Dindo Classification **eTable 2.** Population Characteristics of Study **eFigure 1.** Illustration of the 3 Pathways **eFigure 2.** Illustration of aTTS and iTTS Definitions **eFigure 3.** Illustration of NEST Reclassification by a Clinical Severity Criterion

Supplement 2.
Data Sharing Statement

## References

[1] Gale SC, Shafi S, Dombrovskiy VY, Arumugam D, Crystal JS. The public health burden of emergency general surgery in the United States: a 10-year analysis of the Nationwide Inpatient Sample—2001 to 2010. J Trauma Acute Care Surg. 2014;77(2):202-208. doi:10.1097/TA.0000000000000362 25058242

[2] Endorf FW, Jurkovich GJ. Acute care surgery: a proposed training model for a new specialty within general surgery. J Surg Educ. 2007;64(5):294-299. doi:10.1016/j.jsurg.2007.06.003 17961888

[3] Ogola GO, Gale SC, Haider A, Shafi S. The financial burden of emergency general surgery: national estimates 2010 to 2060. J Trauma Acute Care Surg. 2015;79(3):444-448. doi:10.1097/TA.0000000000000787 26307879

[4] Smith SA, Yamamoto JM, Roberts DJ, . Weekend surgical care and postoperative mortality: a systematic review and meta-analysis of cohort studies. Med Care. 2018;56(2):121-129. doi:10.1097/MLR.0000000000000860 29251716 PMC5770102

[5] Mullen MG, Michaels AD, Mehaffey JH, . Risk associated with complications and mortality after urgent surgery vs elective and emergency surgery: implications for defining “quality” and reporting outcomes for urgent surgery. JAMA Surg. 2017;152(8):768-774. doi:10.1001/jamasurg.2017.0918 28492821 PMC5710495

[6] Havens JM, Peetz AB, Do WS, . The excess morbidity and mortality of emergency general surgery. J Trauma Acute Care Surg. 2015;78(2):306-311. doi:10.1097/TA.0000000000000517 25757115

[7] Schneider C, Tyler LE, Scull EF, Pryle BJ, Barr H. A case-control study investigating factors of preoperative delay in emergency laparotomy. Int J Surg. 2015;22:131-135. doi:10.1016/j.ijsu.2015.08.028 26318501

[8] Cannon CM, Braxton CC, Kling-Smith M, Mahnken JD, Carlton E, Moncure M. Utility of the shock index in predicting mortality in traumatically injured patients. J Trauma. 2009;67(6):1426-1430. doi:10.1097/TA.0b013e3181bbf728 20009697

[9] Norgren L, Hiatt WR, Dormandy JA, ; TASC II Working Group. Inter-Society Consensus for the Management of Peripheral Arterial Disease (TASC II). Eur J Vasc Endovasc Surg. 2007;33(1)(suppl 1):S1-S75. doi:10.1016/j.ejvs.2006.09.024 17140820

[10] Rutherford RB, Baker JD, Ernst C, . Recommended standards for reports dealing with lower extremity ischemia: revised version. J Vasc Surg. 1997;26(3):517-538. doi:10.1016/S0741-5214(97)70045-4 9308598

[11] McIsaac DI, Abdulla K, Yang H, . Association of delay of urgent or emergency surgery with mortality and use of health care resources: a propensity score–matched observational cohort study. CMAJ. 2017;189(27):E905-E912. doi:10.1503/cmaj.160576 28694308 PMC5505757

[12] Leppäniemi A, Jousela I. A traffic-light coding system to organize emergency surgery across surgical disciplines. Br J Surg. 2014;101(1):e134-e140. doi:10.1002/bjs.9325 24272758

[13] Hameed SM, Brenneman FD, Ball CG, ; Canadian Association of General Surgery Committee on Acute Surgery and Critical Care. General surgery 2.0: the emergence of acute care surgery in Canada. Can J Surg. 2010;53(2):79-83. 20334738 PMC2845950

[14] Wanis KN, Hunter AM, Harington MB, Groot G. Impact of an acute care surgery service on timeliness of care and surgeon satisfaction at a Canadian academic hospital: a retrospective study. World J Emerg Surg. 2014;9(1):4. doi:10.1186/1749-7922-9-4 24410769 PMC3892050

[15] Britt RC, Weireter LJ, Britt LD. Initial implementation of an acute care surgery model: implications for timeliness of care. J Am Coll Surg. 2009;209(4):421-424. doi:10.1016/j.jamcollsurg.2009.06.368 19801314

[16] Royal College of Surgeons of England. Separating emergency and elective surgical care: recommendations for practice. 2007. Accessed January 11, 2019. https://www.rcseng.ac.uk/library-and-publications/rcs-publications/docs/seperating-emergency-and-elective/

[17] Kluger Y, Ben-Ishay O, Sartelli M, . World Society of Emergency Surgery study group initiative on Timing of Acute Care Surgery classification (TACS). World J Emerg Surg. 2013;8(1):17. doi:10.1186/1749-7922-8-17 23634784 PMC3652724

[18] Agency for Clinical Innovation. *NSW Emergency Surgery Guidelines and Principles for Improvement*. Government of New South Wales; 2021. Accessed March 12, 2023. https://www1.health.nsw.gov.au/pds/ActivePDSDocuments/GL2021_007.pdf

[19] Cosgrove JF, Gaughan M, Snowden CP, Lees T. Decreasing delays in urgent and expedited surgery in a university teaching hospital through audit and communication between peri-operative and surgical directorates. Anaesthesia. 2008;63(6):599-603. doi:10.1111/j.1365-2044.2008.05441.x 18477270

[20] To KB, Kamdar NS, Patil P, ; Michigan Surgical Quality Collaborative (MSQC) Emergency General Surgery Study Group and the MSQC Research Advisory Group. Acute care surgery model and outcomes in emergency general surgery. J Am Coll Surg. 2019;228(1):21-28.e7. doi:10.1016/j.jamcollsurg.2018.07.664 30359826

[21] Balasubramanian I, Creavin B, Winter D. Impact of an acute surgical unit in appendicectomy outcomes: a systematic review and meta-analysis. Int J Surg. 2018;50:114-120. doi:10.1016/j.ijsu.2017.12.033 29337180

[22] Nagaraja V, Eslick GD, Cox MR. The acute surgical unit model verses the traditional “on call” model: a systematic review and meta-analysis. World J Surg. 2014;38(6):1381-1387. doi:10.1007/s00268-013-2447-1 24430507

[23] Khalil M, Pandit V, Rhee P, . Certified acute care surgery programs improve outcomes in patients undergoing emergency surgery: a nationwide analysis. J Trauma Acute Care Surg. 2015;79(1):60-63. doi:10.1097/TA.0000000000000687 26091315

[24] Ogola GO, Crandall ML, Shafi S. Variations in outcomes of emergency general surgery patients across hospitals: a call to establish emergency general surgery quality improvement program. J Trauma Acute Care Surg. 2018;84(2):280-286. doi:10.1097/TA.0000000000001755 29194319

[25] Légifrance. Code de la santé publique: articles R1123-1 to R1123-19. Accessed March 14, 2023. https://www.legifrance.gouv.fr/codes/article_lc/LEGIARTI000026944947

[26] Peden CJ, Aggarwal G, Aitken RJ, . Guidelines for perioperative care for emergency laparotomy Enhanced Recovery After Surgery (ERAS) Society recommendations: part 1—preoperative: diagnosis, rapid assessment and optimization. World J Surg. 2021;45(5):1272-1290. doi:10.1007/s00268-021-05994-9 33677649 PMC8026421

[27] Saklad M. Grading of patients for surgical procedures. Anesthesiology. 1941;2(3):281-284. doi:10.1097/00000542-194105000-00004

[28] Kalbfleisch JD, Prentice RL. Comparison of survival curves. In: The Statistical Analysis of Failure Time Data. John Wiley & Sons; 1980:16-19.

[29] Koivukangas V, Saarela A, Meriläinen S, Wiik H. How well planned urgency class come true in the emergency surgery? timing of acute care surgery. Scand J Surg. 2020;109(2):85-88. doi:10.1177/1457496919826716 30786828

[30] Murray V, Burke JR, Hughes M, Schofield C, Young A. Delay to surgery in acute perforated and ischaemic gastrointestinal pathology: a systematic review. BJS Open. 2021;5(5):zrab072. doi:10.1093/bjsopen/zrab072 34476466 PMC8413368

[31] Lemma AN, Tolonen M, Vikatmaa P, . Choice of first emergency room affects the fate of patients with acute mesenteric ischaemia: the importance of referral patterns and triage. Eur J Vasc Endovasc Surg. 2019;57(6):842-849. doi:10.1016/j.ejvs.2019.01.002 31126834

[32] Stephens TJ, Peden CJ, Haines R, . Hospital-level evaluation of the effect of a national quality improvement programme: time-series analysis of registry data. BMJ Qual Saf. Published online September 12, 2019. 31515437 10.1136/bmjqs-2019-009537

[33] Peden CJ, Stephens T, Martin G, ; Enhanced Peri-Operative Care for High-risk patients (EPOCH) trial group. Effectiveness of a national quality improvement programme to improve survival after emergency abdominal surgery (EPOCH): a stepped-wedge cluster-randomised trial. Lancet. 2019;393(10187):2213-2221. doi:10.1016/S0140-6736(18)32521-2 31030986

[34] Hall BL, Hamilton BH, Richards K, Bilimoria KY, Cohen ME, Ko CY. Does surgical quality improve in the American College of Surgeons National Surgical Quality Improvement Program: an evaluation of all participating hospitals. Ann Surg. 2009;250(3):363-376. doi:10.1097/SLA.0b013e3181b4148f 19644350

[35] Committee to Develop the Reorganized Specialty of Trauma, Surgical Critical Care, and Emergency Surgery. Acute care surgery: trauma, critical care, and emergency surgery. J Trauma. 2005;58(3):614-616. doi:10.1097/01.TA.0000159347.03278.E1 15761359

[36] Sorelli PG, El-Masry NS, Dawson PM, Theodorou NA. The dedicated emergency surgeon: towards consultant-based acute surgical admissions. Ann R Coll Surg Engl. 2008;90(2):104-108. doi:10.1308/003588408X242042 18325206 PMC2443301

[37] Sarmiento Altamirano D, Himmler A, Chango Sigüenza O, . The successful implementation of a trauma and acute care surgery model in Ecuador. World J Surg. 2020;44(6):1736-1744. doi:10.1007/s00268-020-05435-z 32107595

[38] Mathur S, Lim WW, Goo TT. Emergency general surgery and trauma: outcomes from the first consultant-led service in Singapore. Injury. 2018;49(1):130-134. doi:10.1016/j.injury.2017.09.002 28899559

[39] Earley AS, Pryor JP, Kim PK, . An acute care surgery model improves outcomes in patients with appendicitis. Ann Surg. 2006;244(4):498-504. doi:10.1097/01.sla.0000237756.86181.50 16998358 PMC1856575

[40] Ong MW, Goh SSN, Tung WMJ, . Initial emergency laparotomy outcomes following a transdisciplinary perioperative care pathway in Singapore. Acute Med Surg. 2021;8(1):e702. doi:10.1002/ams2.702 34745640 PMC8552521

[41] Juul S, Kokotovic D, Degett TH, . Validation of the Preoperative Score to Predict Postoperative Mortality (POSPOM) in patients undergoing major emergency abdominal surgery. Eur J Trauma Emerg Surg. 2021;47(6):1721-1727. doi:10.1007/s00068-019-01153-x 31161251

[42] Vester-Andersen M, Lundstrøm LH, Møller MH, Waldau T, Rosenberg J, Møller AM; Danish Anaesthesia Database. Mortality and postoperative care pathways after emergency gastrointestinal surgery in 2904 patients: a population-based cohort study. Br J Anaesth. 2014;112(5):860-870. doi:10.1093/bja/aet487 24520008

[43] Cubas RF, Gómez NR, Rodriguez S, Wanis M, Sivanandam A, Garberoglio CA. Outcomes in the management of appendicitis and cholecystitis in the setting of a new acute care surgery service model: impact on timing and cost. J Am Coll Surg. 2012;215(5):715-721. doi:10.1016/j.jamcollsurg.2012.06.415 22863794

[44] Hoile RW. The National Confidential Enquiry into Peri-operative Deaths (NCEPOD). Aust Clin Rev. 1993;13(1):11-15. 8147765

[45] Levtzion-Korach O, Murphy KG, Madden S, Dempsey C. For urgent and emergent cases, which one goes to the OR first? OR Manager. 2010;26(7):1, 11-13. 20672452

[46] Truskett P. Acute surgery units: the future face of emergency surgery. ANZ J Surg. 2010;80(7-8):477-478. doi:10.1111/j.1445-2197.2010.05371.x 20795954

