# Supplementary material for: Association of Organizational Pathways With the Delay of Emergency Surgery
Source: JAMA Netw Open. 2023 Apr 13;6(4):e238145. doi: 10.1001/jamanetworkopen.2023.8145 (PMC10102875; doi:10.1001/jamanetworkopen.2023.8145)
Supplement: Supplement 1. — eAppendix 1. List of 9 French Centers eTable 1. The Non-Elective Surgery Triage (NEST) Classification eAppendix 2. iTTS of Surgery eAppendix 3. Severity Criteria eAppendix 4. Clavien-Dindo Classification eTable 2. Population Characteristics of Study eFigure 1. Illustration of the 3 Pathways eFigure 2. Illustration of aTTS and iTTS Definitions eFigure 3. Illustration of NEST Reclassification by a Clinical Severity Criterion [file jamanetwopen-e238145-s001.pdf]

Supplementary Online Content

Lepercq D, Gauss T, Godier A, et al. Association of organizational pathways with the delay of emergency surgery. *JAMA Netw Open*. 2023;6(4):e238145. doi:10.1001/jamanetworkopen.2023.8145

eAppendix 1. List of 9 French Centers
eTable 1. The Non-Elective Surgery Triage (NEST) Classification
eAppendix 2. iTTS of Surgery
eAppendix 3. Severity Criteria
eAppendix 4. Clavien-Dindo Classification
eTable 2. Population Characteristics of Study
eFigure 1. Illustration of the 3 Pathways
eFigure 2. Illustration of aTTS and iTTS Definitions
eFigure 3. Illustration of NEST Reclassification by a Clinical Severity Criterion

This supplementary material has been provided by the authors to give readers additional information about their work.

© 2023 Lepercq D et al. *JAMA Network Open*.

eAppendix 1: List of 9 French Centers

Lille University Hospital, Grenoble-Alpes University Hospital, Strasbourg University Hospital, Georges-Pompidou European Hospital, Beaujon Hospital (AP-HP), Henri Mondor Hospital (AP-HP), Lyon Sud Hospital of the Hospices Civils de Lyon (HCL), Edouard Herriot Hospital (HCL), Angers University Hospital.

© 2023 Lepercq D et al. *JAMA Network Open*.

eTable 1: The Non-Elective Surgery Triage (NEST) Classification

Category	**Ideal Time To Surgery (iTTS)**	Possible clinical scenario	**NEST** (Non- Elective Surgery Triage)
**Emergency**	Immediate (< 30 minutes)	Hemodynamic instability (example: bleeding traumatic emergency)	**NEST 1**
	< 1 hour	Viscus perforation, vascular compromise, sepsis (Limb ischemia, diffuse peritonitis)	**NEST 2**
**Urgent**	< 4 hours	Extremity compartment syndrome<br>Ascending cholangitis	**NEST 3**
	< 12 hours	-Bowel obstruction<br>appendicitis	**NEST 4**
**Semi-urgent**	< 48 hours	Second look laparotomy<br>Acute cholescystitis	**NEST 5**
	< 72 hours	First debridement of burn cases	**NEST 6**

© 2023 Lepercq D et al. *JAMA Network Open*.

eAppendix 2: iTTS of Surgery

Min: minutes; Uro: urologic surgery; ORL: otorhinolaryngologist; M/N: muscular and nervous; Hd: hemodynamics; TEA: thromboendateriectomy; ACR: cardio respiratory arrest

EDH: Extradual hematoma, SDE: Subdural hematoma; EVD: external ventricular drainage; Sd: syndrome, OPH: ophthalomologist

			Category					
			Emergency		Urgent		Semi Urgent	
Speciality	Surgery	Patient	NEST 1	NEST 2	NEST 3	NEST 4	NEST 5	NEST 6
iTTS	Note that a patient with severity criteria can change NEST category		< 30 min	< 1H	< 4H	< 12H	< 48H	< 72H
	Infectious emergency			NEST 2				
	Hemorrhagic emergency			NEST 2				
Digestive	Liver transplant					< 8h if M3		
	Pancreas transplant					NEST 4		
	Appendicectomy					NEST 4		
	Occlusive syndrome					NEST 4		
	cholecystectomy						NEST 5	
	Stranguled hernia				NEST 3			
	Abcess						NEST 5	
	Mesenteric ischemia			NEST 2				
	digestive perforation / vascular emergency			NEST 2				
Orthopaedics	Hip fracture	Closed fracture					NEST 5	
	Pelvic fracture	Tile B ou C / closed		Open		closed		
	Femur fracture	closed				NEST 4		
		Classification Gustilo III ou IV			NEST 3			
	Malleolar fracture	closed					NEST 5	
		Classification Gustilo III ou IV			NEST 3			
	tibia/fibula fracture	closed					NEST 5	
		Classification Gustilo III ou IV			NEST 3			
	Wrist fracture	closed						NEST 6
		Classification Gustilo III ou IV			NEST 3			

© 2023 Lepercq D et al. *JAMA Network Open*.

Speciality	Surgery	Patient	Emergency: NEST 1	Emergency: NEST 2	Urgent: NEST 3	Urgent: NEST 4	Semi Urgent: NEST 5	Semi Urgent: NEST 6
iTTS			< 30 min	< 1H	< 4H	< 12H	< 48H	< 72H
Uro	testicular torsion			NEST 2				
	uretheral probe			septic shock		hyperalgesic		
	Renal transplantation						< 18H si M3	
Maxillofacial/plastic sur	Abcess/dental cellulitis				NEST 3			
	Wound trimming and suturing				NEST 3			
	Osteosynthesis mandible	without entrapment M/N				NEST 4		
		with entrapment M/N			NEST 3			
	Intermaxillary blocking + osteosynthesis					NEST 4		
	Osteosynthesis orbital floor	without entrapment M/N						NEST 6
		with entrapment M/N			NEST 3			
	Flap revision				NEST 3			
ORL	Phlegmon/tonsil abcess					NEST 4		
	tracheotomy	respiratory/ Hd distress	NEST 1					
Vascular - Thoracic - Heart surgery	abdominal aortic aneuvrysm	Fissure syndrome			NEST 3			
		Ruptured	NEST 1					
	Acute limb ischemia			Rutherford>2a				
	Exploratory thoracotomy		if ACR					
	carotid TEA						NEST 5	
	coronary aortic bypass surgery				NEST 3	unstable angina		
	Tamponade		NEST 1					
	Valvuloplasty	instability Hd	NEST 1			embolism endocarditis		
	Aortic dissection		NEST 1					
	Aortic isthmus rupture			NEST 2				
	Trauma + bronchial wound		NEST 1					
	Trauma + vascular wound		NEST 1					
	Heart transplant				NEST 3			
	lung transplant					NEST 4		

Speciality	surgery	Patient	Emergency: NEST 1	Emergency: NEST 2	Urgent: NEST 3	Urgent: NEST 4	Semi Urgent: NEST 5	Semi Urgent: NEST 6
iTTS			< 30 min	< 1H	< 4H	< 12H	< 48H	< 72H
	Multiple organ harvest				NEST 3			
Neurosurg	Evacuation EDH		NEST 1					
	Evacuation SDH		NEST 1					
	installation of EVD/hydrocephaly			NEST 2				
	Spine fixation				NEST 3			
OPH	Intravitreal injection					NEST 4		
	Eye injury				NEST 3			
Gyneco	Extra uterine pregnancy			NEST 2			NEST 5	
	Adnexal torsion			NEST 2				
	Abcess / phlegmon				NEST 3			
	Endo uterine aspiration			NEST 2				
Hand surge	Wound exploration				if devascu or complex wound	without devascularisation <24h		
	Surgical debridement				severe sepsis	phlegmon		
	Osteosynthesis	Closed			If compartement Sd	fracture + luxation		NEST 6
		Open			NEST 3			

© 2023 Lepercq D et al. *JAMA Network Open*.

eAppendix 3: Severity Criteria

A total of five severity criteria were defined:

1) Infectious: septic shock with at least two of the following Sepsis-related Organ Failure Assessment (SOFA) criteria present [45]:

- Sepsis-related hypotension (blood pressure (BP) below 90/60 mmHg or mean arterial pressure (MAP) below 60 mmHg).
- Lactate above normal laboratory values.
- Diuresis less than 0.5 mL/kg/hr for more than two hours despite adequate filling.
- Arterial oxygen pressure on inspired oxygen fraction less than 250 mmHg in the absence of lung disease.
- Creatinine levels greater than 2 mg/dL (176.8 mmol/L).
- Bilirubin level greater than 2 mg/dL (34.2 mmol/L).
- Thrombocytopenia less than 100,000 mm3.

2) Hemorrhagic: hemorrhagic shock with the presence of the following four criteria for hemorrhagic emergency [46]:

- Active bleeding.
- MAP less than 60 mmHg with or without Noradrenaline.
- Shock Index (SI = Heart rate (beats/minutes, bpm) over systolic blood pressure (mmHg) greater than 0.9 (8).
- Extravasation of contrast medium during a CT scan.

3) Ischemic: acute limb ischemia with the presence of the following two criteria for ischemic urgency [9, 10]:

- Moderate (Rhuterford category IIb) to complete (Rhuterford category III) motor and sensory deficit.
- Inaudible arterial and audible venous Doppler.

4) Neurological: presence of one of the following neurological emergency criteria [47]:

- Glasgow Coma Scale (GCS) score less than 13 or a loss of two points on the Glasgow Coma Scale since the initial assessment.
- Incomplete or progressive neurological deficit with unstable injury or spinal cord compression
- Symptomatic extra dural hematoma.
- Subdural haematoma greater than 5 mm and midline deviation greater than 5 mm
- Acute hydrocephalus.

5) Polytrauma: patient meeting the following definition:

© 2023 Lepercq D et al. *JAMA Network Open*.

"Severe trauma with several physical injuries, at least one of which is life-threatening in the very short term" [48].

[45] Montravers P, Dupont H, Leone M, Constantin JM, Mertes PM, Laterre PF et al. Prise en charge des infections intra-abdominales. *Anesth Réanimation* 2015; 1(1): 75–99.

[46] Duranteau J, Asehnoune K, Pierre S, Ozier Y, Leone M, Lefrant JY. Recommandations sur la réanimation du choc hémorragique. *Anesth Réanimation* 2015; 1(1): 62–74.

[47] Geeraerts T, Velly L, Abdennour L, Asehnoune K, Audibert G, Bouzat P et al. Prise en charge des traumatisés crâniens graves à la phase précoce (24 premières heures). *Anesth Réanimation* 2016; 2(6): 431–453.

[48] Laplace C, Duranteau J. Accueil du polytraumatisé. 51e *Congrès national d'anesthésie et de réanimation. Médecins. Les essentiels* 2009 :13.

© 2023 Lepercq D et al. *JAMA Network Open*.

eAppendix 4: Clavien-Dindo Classification

Grade	
1	Any deviation from the normal postoperative course without the need for pharmacological treatment or surgical, endoscopic and radiological interventions.<br>Acceptable therapeutic regimens are: drugs as antiemetics, antipyretics, analgetics, diuretics and electrolytes and physiotherapy.<br>This grade also includes wound infections opened at the bedside.
2	Requiring pharmacological treatment with drugs other than such allowed for grade I complications. Blood transfusions, antibiotics and total parenteral nutrition are also included.
3	Requiring surgical, endoscopic or radiological intervention
3a	Intervention under regional/local anesthesia
3b	Intervention under general anesthesia
4	Life-threatening complication requiring intensive care/intensive care unit management
4a	Single organ dysfunction
4b	Multi-organ dysfunction
5	Patient demise

© 2023 Lepercq D et al. *JAMA Network Open*.

eTable 2: Population Characteristics of Study

	All patients	DET	DOR	NOR
	n = 1144	n = 649 (57%)	n = 320 (28%)	n = 171 (15%)
**Age**, years, mean (SD)	54.8 (21.1)	55.0 (21.8)	54.6 (20.4)	54.8 (19.5)
**Female sex, n (%)**	459 (40%)	251 (39%)	146 (46%)	59 (35%)
**Male sex, n (%)**	685 (60%)	398 (61%)	174 (54%)	112 (65%)
ASA score				
n	1144	648	319	168
I	356 (31%)	212 (32.7%)	96 (30.1%)	46 (27.4%)
II	365 (32%)	215 (33.2%)	107 (33.5%)	42 (25%)
III	310 (27%)	172 (26.5%)	82 (25.7%)	55 (32.7%)
IV	77 (7%)	33 (5.1%)	27 (8.5%)	17 (10.1%)
V	31 (3%)	16 (2.5%)	7 (2.2%)	8 (4.8%)
Severity criteria				
n	1144	649	310	168
Infectious	60 (5.3%)	36 (5.5%)	16 (5.2%)	8 (4.8%)
Hemorrhagic	40 (3.5%)	24 (3.7%)	5 (1.6%)	11 (6.5%)
Ischaemic	29 (2.6%)	16 (2.5%)	8 (2.6%)	5 (3.0%)
Neurological	76 (6.7%)	28 (4.3%)	24 (7.7%)	24 (14.3%)
Polytrauma	34 (3.0%)	14 (2.2%)	10 (3.2%)	10 (6.0%)
No Criteria	888 (78.9%)	531 (81.8%)	247 (79.7)	110 (65.5%)
Theory NEST				
n	799	439	243	115
NEST 1	72 (9.0%)	40 (9.1%)	12 (4.9%)	20 (17.4%)
NEST 2	88 (11%)	32 (7.3%)	39 (16.0%)	17 (14.8%)
NEST 3	100 (12.2%)	47 (10.7%)	31 (12.8%)	22 (19.1%)
NEST 4	212 (26.5%)	117 (26.7%)	60 (24.7%)	35 (30.4%)
NEST 5	269 (33.7%)	166 (37.8%)	83 (34.2%)	19 (16.5%)
NEST 6	58 (7.3%)	37 (8.4%)	18 (7.4%)	2 (1.7%)
No NEST identified a priori	345	210	77	56

© 2023 Lepercq D et al. *JAMA Network Open*.

	All patients	DET	DOR	NOR
NEST of surgeon				
n	984	508	303	170
NEST 1	58 (5.9%)	32 (6.3%)	9 (3%)	17 (10%)
NEST 2	79 (8%)	26 (5.1%)	40 (13.2%)	13 (7.6%)
NEST 3	174 (17.7%)	87 (17.1%)	61 (20.1%)	25 (14.7%)
NEST 4	250 (25.4%)	132 (26%)	71 (23.4%)	47 (27.6%)
NEST 5	305 (31%)	171 (33.7%)	83 (27.4%)	49 (28.8%)
NEST 6	118 (12%)	60 (11.8%)	39 (12.9%)	19 (11.2%)
Working period				
n	1144	646	319	170
Working hours, n (%)	631 (55.4%)	351 (54.3%)	162 (50.8%)	117 (68.8%)
Time outside working hours, n (%)	508 (44.6%)	295 (45.7%)	157 (49.2%)	53 (31.2%)

Values are expressed as numbers (percentage), unless otherwise indicated; *DET: dedicated emergency theatre, DOR: dedicated emergency operating room, NOR: absence of a dedicated emergency operating room, ASA: American society of anesthesiologists, NEST: Non-Elective Surgery Triage.*

© 2023 Lepercq D et al. *JAMA Network Open*.

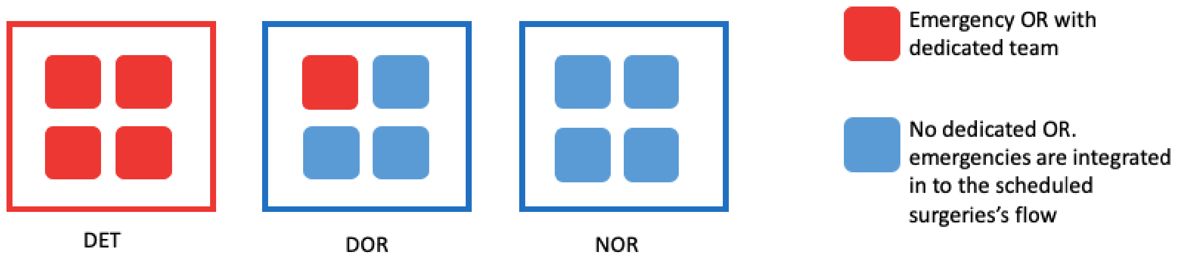

<u>eFigure 1:</u> Illustration of the three pathways. *DET: dedicated emergency theatre, DOR: dedicated emergency operating room, NOR: absence of a dedicated emergency operating room.*

© 2023 Lepercq D et al. *JAMA Network Open*.

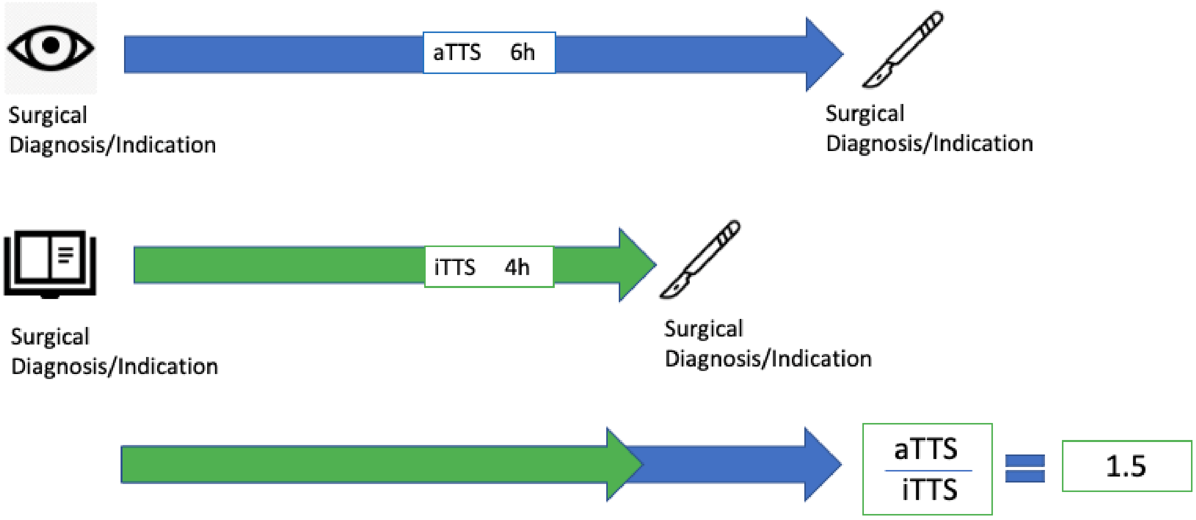

<u>eFigure 2:</u> Illustration of aTTS and iTTS definitions. *aTTS: actual time to surgery, iTTS: ideal time to surgery.*

© 2023 Lepercq D et al. *JAMA Network Open*.

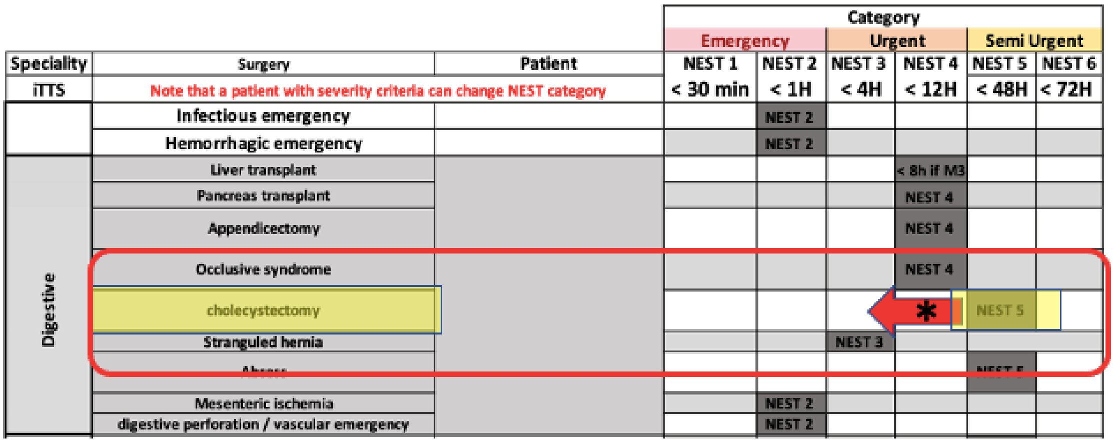

* Cholecystectomy for acute cholecystitis is considered NEST 5 category
Presence of clinical severity criteria, like shock allows reclassification to e.g. NEST 4 or NEST 3

<u>eFigure 3:</u> Illustration of NEST reclassification by a clinical severity criterion. *aTTS: actual time to surgery, iTTS: ideal time to surgery.*

© 2023 Lepercq D et al. *JAMA Network Open*.
