# Supplementary material for: Association of Organizational Pathways With the Delay of Emergency Surgery
Source: JAMA Netw Open. 2023 Apr 13;6(4):e238145. doi: 10.1001/jamanetworkopen.2023.8145 (PMC10102875; doi:10.1001/jamanetworkopen.2023.8145)
Supplement: Supplement 2. — Data Sharing Statement [file jamanetwopen-e238145-s002.pdf]

Data Sharing Statement

Lepercq. Association of Organizational Pathways With the Delay of Emergency Surgery. *JAMA Netw Open*. Published April 13, 2023. doi:10.1001/jamanetworkopen.2023.8145

Data
Data available: Yes
Data types: Deidentified participant data
How to access data: Emeline.cailliau@chu-lille.fr
When available: With publication

Supporting Documents
Document types: Statistical/analytic code
How to access documents: Emeline.cailliau@chu-lille.fr
When available: With publication

Additional Information
Who can access the data: Anyone requesting the data
Types of analyses: For specified purpose
Mechanisms of data availability: with a signed data access agreement
